# An Automated Power Evaluation Workbench for Triboelectric Nanogenerators

**DOI:** 10.3390/mi13030444

**Published:** 2022-03-15

**Authors:** Ming Yuan, Chunhui Li, Sheng Zhang, Yannan Xie

**Affiliations:** 1 College of Automation & Artificial Intelligence, Nanjing University of Posts and Telecommunications, Nanjing 210023, China; lichunhuiaynu@163.com; 2 Suzhou Acoustic Technology Institute Co., Ltd., Changshu 215500, China; shengzhang@iat-center.com; 3 State Key Laboratory of Organic Electronics and Information Displays, Institute of Advanced Materials (IAM), Nanjing University of Posts and Telecommunications, Nanjing 210023, China

**Keywords:** triboelectric nanogenerator, automation test, resistance synthesis, power evaluation

## Abstract

Triboelectric nanogenerators (TENGs) have high potential in self-powered sensing and energy harvesting applications. In general, TENGs’ internal source resistance is high, and their output power varies under different load resistance values. Therefore, a resistance box is required to evaluate their energy harvesting performance and obtain the power curve under different load values. The load tuning process is usually performed by hand. This repetitive process is time-consuming and error-prone. Consequently, an Automated Power Evaluation Workbench (APEW) is developed, making the resistance switching and power measuring process program-controlled. The resistance value is resolved using the Octal decomposition principle. In addition, a resistance synthesis algorithm is proposed to alter the resistance value with a minimum step of 1 Ohm. The target resistance value is physically synthesized by relay switching, while digital lines control the relays. The proposed APEW is then evaluated experimentally, and the obtained results are compared with those of the traditional manual switching approach. It is deduced that the two power curves are almost identical. Therefore, it is believed that the proposed APEW will play a crucial role in TENG’s further development.

## 1. Introduction

The triboelectric nanogenerator (TENG) uses triboelectrification and electrostatic induction mechanisms that convert several environmental energies into electricity, such as vibration, fluid, kinetic and acoustic [1,2,3,4,5]. TENGs can scavenge with high entropy energy and provide electrical power to several low-power consumption electronic devices with proper design. These devices’ numbers can reach billions in the Internet of Things (IoT) era [6]. In addition to energy harvesting functionality, the TENG can also be used as a self-powered sensor, which can be used for sport [7], pulmonary [8], configurational [7], wind [9], and motion [10] sensing applications.

The TENG has high voltage, low current, and high internal impedance properties. These attributes make the corresponding measurement and instrumentation development essential in the research and development period. 

The low current value can be measured using a feedback or shunt ammeter. In general, the feedback ammeter has a better resolution and less burden voltage than the shunt ammeter. For instance, the low-noise current amplifier SR570 is widely used in the TENG’s short circuit measurement [11,12,13]. A low-cost current preamplifier is developed in [10]. It shows identical current waveforms compared with the SR570 approach [14]. The TENG’s open-circuit voltage can be measured using an electrometer, owing to its input resistance reaching up to T-Ohm level [15,16,17,18]. Another voltage measurement approach uses the oscilloscope/digital multimeter and high voltage probe, which allows a high-speed and large-amplitude voltage measurement [19,20,21,22]. Hong et al. [23] propose a testbed for the contact-separation-type TENG to precisely and quantitatively benchmark the TENG’s performance. They then develop another testbed suitable for the rotational TENG’s performance evaluation [24]. Sharma et al. [25] propose a comprehensive test workbench, which allows the discovery of TENG’s performance in an affordable approach.

In addition, it is always essential to find the TENG’s output power curve versus different load resistance values, which helps us to evaluate the TENG’s performance intuitively. The resistance tuning and power tracking processes are tedious. However, they are highly repeatable. Therefore, this task can be automatically fulfilled, which has not been reported yet.

In this paper, a computer-controlled Automated Power Evaluation Workbench (APEW) is designed and developed to obtain the TENG’s power curve automatically. A resistance schedule algorithm is also proposed, making the synthesized resistance value with the minimum change step of 1 Ohm. Physically, the resistance value is scheduled and controlled by relays. The synthesized resistance value is formed with proper tuning of the relays’ ON/OFF status, which is governed by the resistance schedule algorithm.

## 2. Resistance Synthesis Principle and Development 

### 2.1. Resistance Decomposing and Synthesis

The TENG’s principle consists of coupling contact electrification and electrostatic induction, with inherent capacitance properties. The total voltage difference between the two electrodes can be expressed as [26]:

(1) $$V=-\frac{1}{C(x)}Q+V_{OC}(x)$$

where x is the distance between the two electrodes, Q represents the transferred charges, C(x) denotes the TENG’s inherent dynamic capacitance, and $V_{OC}(x)$ is the TENG’s open-circuit voltage. 

Therefore, the equivalent circuit model of TENG has a serial connection of an ideal voltage source with a variable capacitor. 

**Figure 1:**
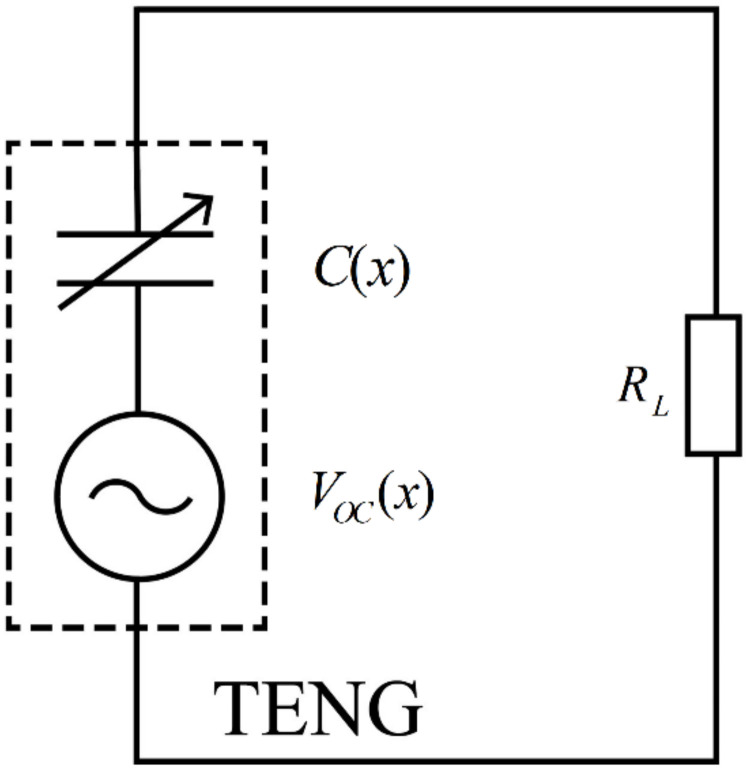
Schematic diagram of the TENG.

The TENG’s equivalent circuit model is presented in Figure 1. The TENG’s internal impedance is $Z_{S}=1/j\omega C(x)$, its external load resistance is denoted by $R_{L}$, and the load voltage is represented by $V_{L}$. 

The detailed expression of $V_{L}$ and the consumption power $P_{L}$ can be expressed as:

(2) $$V_{L}=\frac{R_{L}V_{OC}(x)}{R_{L}+\frac{1}{j\omega C(x)}}$$

(3) $$P_{L}=\frac{V_{OC}^{2}(x)}{R_{L}}{\left(\frac{R_{L}}{R_{L}+\frac{1}{j\omega C(x)}}\right)}^{2}$$

Hence, the TENG’s output power is highly related to the external load resistance $R_{L}$. In order to automatically obtain the TENG’s output power curve, several external resistance values should be synthesized in a well-organized manner. However, the number of resistors should be limited to ensure that the measurement system is physically realizable.

The synthesized resistance $R_{synthesized}$ (integral value) is designed using the Octal decomposition principle. The target resistance value is the resistor ($R_{ij}$) summation from N different columns, where each column comprises eight rows. Each column only allows one element to feed through the circuit at one time. The $R_{synthesized}$ value is given by:

(4) $$R_{synthesized}=\sum_{j=1}^{N}R_{ij}=\sum_{j=1}^{N}i\cdot 8^{j-1}(i=0,\ldots \ldots ,7)$$

where i represents the row number, j denotes the column number, and $R_{ij}$ is the corresponding resistance value which follows the Octal decomposition principle. 

**Figure 2:**
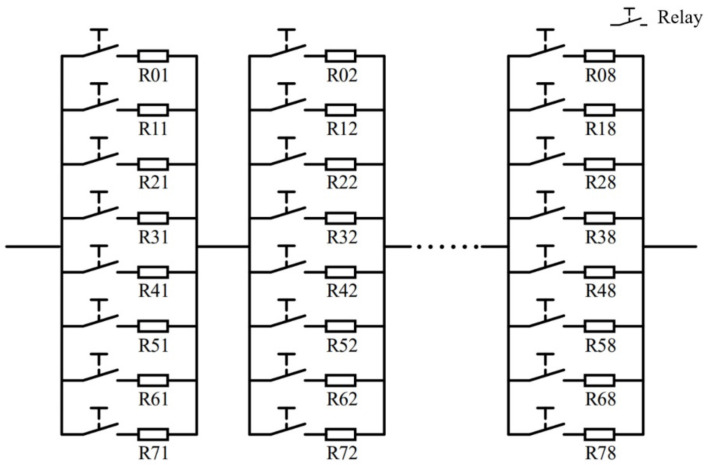
Schematic diagram of the resistance synthesis using the Octal decomposition principle.

For instance, considering that the resistors are divided into eight columns, each column comprises eight parallel resistors, and a relay accompanies each resistor, the schematic diagram of the resistance synthesis is presented in Figure 2. 

Only one relay in each column is allowed to be closed at one time, and the synthesized resistance is the summation of the eight resistors from the eight corresponding columns. The resistance values in the jth column should satisfy $R_{ij}=i\cdot 8^{j-1}(i=0,\ldots \ldots ,7)$. The calculated resistance values are presented in Table 1.

Considering that a large portion of the calculated theoretical resistance values is not available from commercial products, the used resistance values for system realization are presented in Table 2. The selecting rule is that the resistance value for implementation is smaller than the theoretical value. However, it is the closest to it. In addition, the synthesized element comprises three resistors placed in series.

### 2.2. Resistance Schedule Algorithm

In order to evaluate the TENG’s output power, the resistance value should be changed in a programmable manner. Therefore, a scheduling algorithm able to form specific resistance values according to the Octal decomposition principle and provide switching signals to the corresponding relays is required. When switching signals are generated, specific relays are closed, which indicates that the accompanying resistor becomes one part of the synthesized resistance.

For instance, the resistance values shown in Table 2 are used to form the desired resistance value. In the proposed schedule algorithm, Column 8 is the start searching point. Compared with the desired resistance value, the smallest and closest element is selected in this column. The selected element subtracts the desired resistance value in Column 8 and searches the smaller and closest element given in Column 7. This process continues until the resistor shown in Column 1 is determined. For instance, if the desired resistance is 240 kΩ, after this algorithm computation, the obtained composed element values are 4 Ω for Column 1, 24 Ω for Column 2, 320 Ω for Column 3, 2.56 kΩ for Column 4, 8.192 kΩ for Column 5, 228.9 kΩ for Column 6, 0 Ω for Column 7 and 0 Ω for Column 8. 

**Figure 3:**
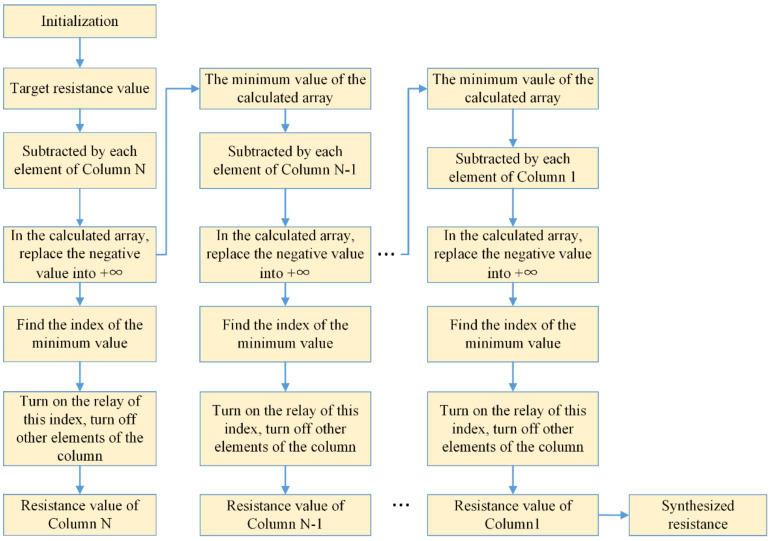
Flowchart of the resistance schedule algorithm.

The detailed flowchart of the resistance schedule algorithm is presented in Figure 3.

## 3. APEW Realization

**Figure 4:**
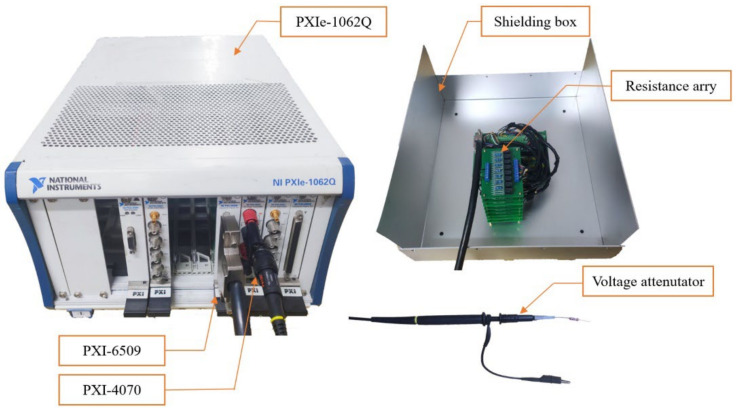
The APEW components.

Relays (Omron G5V-1 DC5V), carbon-film resistors (1% accuracy), a PXI-6509 digital IO module [27], and a PXI-4070 digital multimeter module [28] are selected as the APEW components (Figure 4).

A voltage attenuator is built up to minimize the impedance loading effect and allow high voltage measurement capacities. The attenuator’s internal resistance is 530 MΩ, which achieves 530:1 voltage attenuation when the input resistance of the PXI-4070 is set to 1 MΩ. Note that the actual voltage is re-scaled in the software. 

The programable resistance array is connected to the TENG in series. It is controlled by the PXI-6509 DIO module, which is equipped with 96 DIO lines to drive the relays. When the status of the digital line is set to “ON”, a DC 5 V signal is generated at the digital output line, which brings an electromagnetic force to operate the switch. This results in linking the subsequent resistor of the relay to the measurement circuit. When the status of the digital line is set to “OFF”, a DC 0 V signal is generated at the digital output line. Consequently, the relay disconnects the resistor from the measurement circuit.

The resistors are formed by modules, which are presented in Table 2. When the synthesized resistance is generated, its voltage will be scaled by the voltage attenuator. This voltage value is acquired by the PXI-4070 module and then processed by the LabVIEW software.

## 4. Experiment Verification

### 4.1. Synthesized Resistance Verification

**Figure 5:**
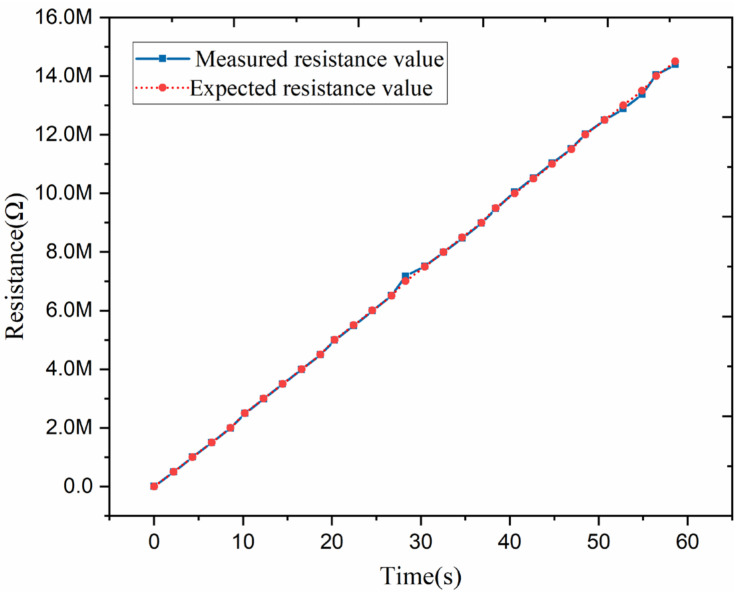
The synthesized resistance measurement data.

It is essential to verify the synthesized resistance values’ correctness before performing the power evaluation step. In the test case, the initial resistance value, final resistance value, and resistance value change are set to 0 MΩ, 14 MΩ, and 0.5 MΩ per two seconds, respectively. The PXI-4070 multimeter is used to measure the synthesized resistance. The measured data are presented in Figure 5. The test data indicate that the resistance can be programmably synthesized in the desired manner and used for TENG power evaluation.

### 4.2. Power Evaluation 

**Figure 6:**
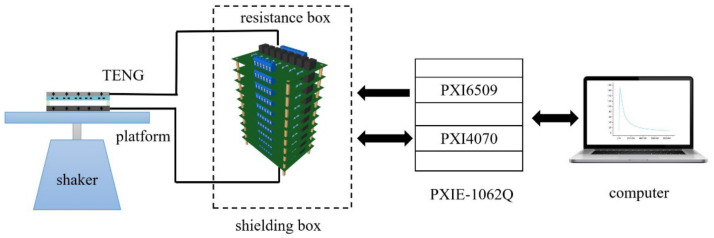
Schematic diagram of the APEW system.

A TENG device is placed on a vibration shaker, generating a contact-separation movement under harmonic vibration excitation. The APEW is connected to the TENG in series. A metallic box shields the resistance circuit boards to prevent electromagnetic interference. The PXI-6509 module is used to control the relays according to the proposed algorithm. In addition, the PXI-4070 module is used to acquire the voltage waveform across the synthesized resistor. The schematic diagram of the experimental power evaluation system is illustrated in Figure 6.

**Figure 7:**
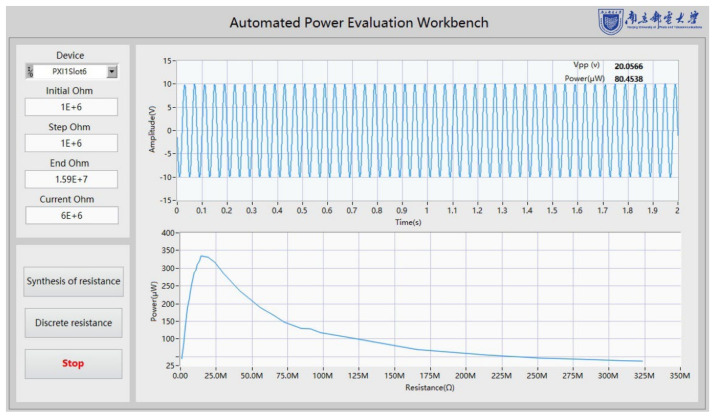
The APEW control panel.

The software interface is presented in Figure 7. After a complete round, the power curve can be automatically generated and shown in the front panel of the software.

The “Initial Ohm” sets the minimum load resistance value during the test, while the synthesized resistance increases according to the “ Step Ohm” value. When the “Synthesis of resistance” is pressed, the APEW starts to work. Considering the resistor’s accuracy and accelerating measurement speed, when the synthesized resistance value is greater than 15 MΩ, 16 fixed large-value resistors are used as external load. Consequently, a quicker test process is performed when the “Discrete resistance” button is pressed, covering the fixed large load resistance values. The collected voltage waveform is displayed in a real-time waveform diagram. When the test is completed, the output power curve of TENG is automatically generated in the waveform chart.

**Figure 8:**
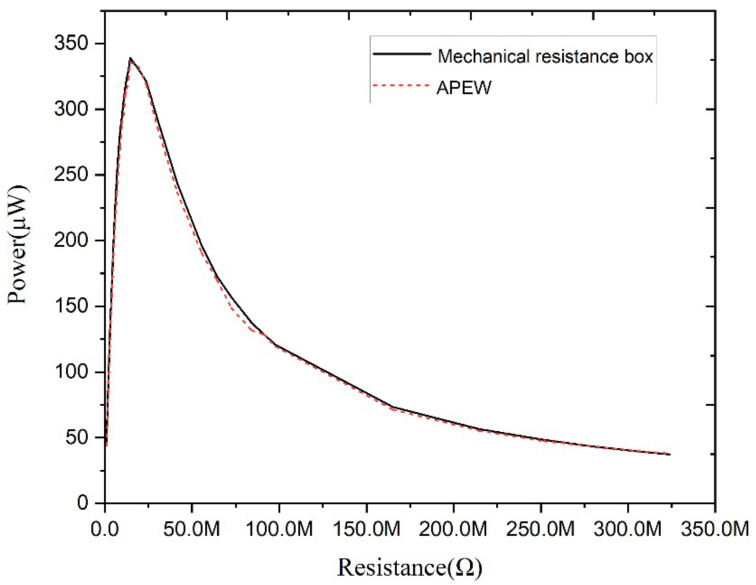
The test results of the TENG output power.

The power curve obtained by APEW is also compared with the power curve using the mechanical resistance box approach, which is a tuning process performed manually. These two curves are shown in Figure 8.

It can be seen from the measured data of the APEW system that the TENG’s maximum output power is 335.82 μW and the optimal load resistance is 14.587 MΩ. On the contrary, the manual tuning’s maximum output power and optimal matching load are 338.89 μW and 14.587 MΩ, respectively. Therefore, the power deviation is 0.91%, which is due to the accuracy of the electronic components and variation om the parasitic parameters. Consequently, the experimental data justify the feasibility of using APEW to obtain the TENG’s output power curve.

## 5. Conclusions

This paper proposes a workbench to achieve automatic power evaluation for the TENG. The arbitrary integral resistance value can be synthesized using the Octal decomposition. The resistance value increases by the preset step size when the program starts. The TENG’s output power can be automatically measured for different resistance values, followed by the resistance schedule algorithm. After the test is completed, the calculated power values are saved, and the TENG’s output power curve is generated. This APEW system reduces human intervention, operators’ workload, and error probability. Finally, all the components are modularly designed, guaranteeing flexibility, expandability, and maintainability.

## Figures and Tables

**Table 1:** $R_{ij}$ theoretical resistance values.

$R_{ij}(\Omega )$	*j = 1*	*j = 2*	*j = 3*	*j = 4*	*j = 5*	*j = 6*	*j = 7*	*j = 8*
***i = 0***	0	0	0	0	0	0	0	0
***i = 1***	1	8	64	512	4.096 k	32.768 k	262.144 k	2.097152 M
***i = 2***	2	16	128	1.024 k	8.192 k	65.536 k	524.288 k	4.194304 M
***i = 3***	3	24	192	1.536 k	12.288 k	98.304 k	786.432 k	6.291456 M
***i = 4***	4	32	256	2.048 k	16.384 k	131.072 k	1.048576 M	8.388608 M
***i = 5***	5	40	320	2.56 k	20.48 k	163.84 k	1.31072 M	10.485760 M
***i = 6***	6	48	384	3.072 k	24.576 k	196.608 k	1.572864 M	12.582912 M
***i = 7***	7	56	448	3.584 k	28.672 k	229.376 k	1.835008 M	14.680064 M

**Table 2:** $R_{ij}$ Resistance values used for implementation.

$R_{ij}(\Omega )$	*j = 1*	*j = 2*	*j = 3*	*j = 4*	*j = 5*	*j = 6*	*j = 7*	*j = 8*
***i = 0***	0	0	0	0	0	0	0	0
***i = 1***	1	8	64	512	4.096 k	32.7 k	261.6 k	2 M
***i = 2***	2	16	128	1.024 k	8.192 k	65.4 k	523.2 k	4 M
***i = 3***	3	24	192	1.536 k	12.288 k	98.1 k	784.8 k	6 M
***i = 4***	4	32	256	2.048 k	16.384 k	130.8 k	1.046 M	8 M
***i = 5***	5	40	320	2.56 k	20.48 k	163.5 k	1.308 M	10 M
***i = 6***	6	48	384	3.072 k	24.576 k	196.2 k	1.5696 M	12 M
***i = 7***	7	56	448	3.584 k	28.672 k	228.9 k	1.8312 M	14 M
